# Young Adult ADHD Symptoms in the General Population and Neurocognitive Impairment

**DOI:** 10.1177/10870547231201870

**Published:** 2023-10-21

**Authors:** Sharifah Shameem Agha, Lucy Riglin, Rhian Carbury, Rachel Blakey, Amy Shakeshaft, Ajay K. Thapar, Kate Tilling, Stephan Collishaw, Evie Stergiakouli, Anita Thapar, Kate Langley

**Affiliations:** 1 Division of Psychological Medicine and Clinical Neurosciences, MRC Centre for Neuropsychiatric Genetics and Genomics, Cardiff University, UK; 2 Cwm Taf Morgannwg University Health Board, Wales, UK; 3 Wolfson Centre for Young People’s Mental Health, Cardiff University, UK; 4 MRC Integrative Epidemiology Unit, University of Bristol, UK; 5 School of Psychology, Cardiff University, UK

**Keywords:** ADHD symptoms, adulthood, neurocognitive impairment, ALSPAC

## Abstract

**Objective:**

Neurocognitive impairments are associated with child and adult ADHD in clinical settings. However, it is unknown whether adult ADHD symptoms in the general population are associated with the same pattern of cognitive impairment. We examined this using a prospective, population-based cohort spanning birth to age 25 years.

**Methods:**

We examined associations between self-reported adult ADHD symptoms and cognitive task performance (attention and response inhibition) in adulthood and childhood.

**Results:**

Self-rated ADHD symptoms at age 25 were associated with poorer performance in age 25 cognitive tasks capturing ADHD-related functioning (attention *B* = −0.03, 95% CI [0.05, −0.01], *p* = .005; response inhibition *B* = −0.03, 95% CI [−0.05, −0.01], *p* = .002).

**Conclusions:**

Neurocognitive impairments linked to adult ADHD symptoms in the general population, are similar to those found in people with childhood ADHD symptoms and are consistent with findings in adult ADHD clinical samples.

## Introduction

Attention Deficit Hyperactivity Disorder (ADHD) is a neurodevelopmental condition that typically onsets in childhood and is characterized by impairing levels of inattention and/or excessive motor activity and impulsivity. ADHD also affects adults with a prevalence of approximately 2.5% (Simon et al., 2009).

Research suggests that there are differences in ADHD in adulthood compared to ADHD in childhood. For example, adults with ADHD show fewer hyperactivity symptoms than children (Faraone et al., 2006). Research also indicates the male preponderance observed in childhood ADHD is reduced in adult ADHD (Biederman et al., 1994; Faraone et al., 2015)

ADHD has long been associated with neurocognitive deficits and several theories and pathways have been proposed to explain the neurocognitive mechanisms underlying ADHD (Barkley, 1997; Castellanos et al., 2006; Pennington & Ozonoff, 1996; Sonuga-Barke et al., 2010). Extensive research in children with ADHD has observed neurocognitive deficits in multiple domains including attentional and inhibitory processes as well as other domains of executive functioning like processing speed and reward dysregulation (Coghill, Seth, & Matthews, 2014; Huang-Pollock et al., 2012; Seidman, 2006; Willcutt et al., 2005). The neuropsychological profile of ADHD is heterogeneous with effect sizes of associations ranging from small to moderate, and not all children show neurocognitive deficits (Willcutt et al., 2005). Research suggests that while cognitive performance may improve over time for both children with ADHD and controls (from childhood to late adolescence), the difference between those with ADHD and controls persists (Lin & Gau, 2019; Seidman, 2006).

Studies of adults with a clinical diagnosis of ADHD generally report that the pattern of neurocognitive deficits is similar to that found in children with ADHD; small to moderate effect sizes across multiple cognitive domains, with notable impairments relative to controls in attention, response inhibition and working memory (Boonstra et al., 2005; Coghill, Hayward et al., 2014; Hervey et al., 2004; LeRoy et al., 2019; Mostert et al., 2015; Pievsky & McGrath, 2018; Schoechlin & Engel, 2005; Sonuga-Barke et al., 2010). Similarly there is also heterogeneity in adult ADHD such that not all those with the condition show neurocognitive deficits (Mostert et al., 2015). However there is some evidence that the neuropsychological profile in adults may differ between inattentive and hyperactive-impulsive subtypes of ADHD (Barkley, 1997), although others have also found that there are no differences between ADHD subtypes (Murphy et al., 2001) whilst a systematic review has noted mixed finding but that there are few studies which allow direct comparisons, especially in the analysis of the hyperactive-impulsive subtype (LeRoy et al., 2019).

Attentional processes have long been considered as an important neurocognitive domain associated with ADHD (Douglas & Peters, 1979). In particular, adults with ADHD or persisting ADHD symptoms tend to report more inattention difficulties than hyperactivity and impulsivity, indicating inattention as a prominent feature in adult ADHD (Biederman et al., 2009; Wilens et al., 2009). Research has shown that adults with ADHD show difficulties in a variety of attentional processes (Schoechlin & Engel, 2005; Tucha et al., 2017). Sustained attention, which is the ability to maintain focus on one or more sources of information over time, is one attentional process which is important for successful daily functioning (Marchetta et al., 2008; Mirsky et al., 1999; Tucha et al., 2017). Another core domain underlying neurocognitive deficits in ADHD is response inhibition (Barkley, 1997). Response inhibition can be defined as the ability to deliberately suppress or interrupt the expression of responses (cognitive, emotional and behavioral) (Coutinho et al., 2017; Schachar et al., 2000). Previous research on clinical samples of adults with ADHD show evidence of difficulties in both attention and response inhibition compared to controls (Avisar & Shalev, 2011; Marchetta et al., 2008; Salomone et al., 2020; Tucha et al., 2017).

Previous research shows that ADHD symptom scores behave as a continually distributed trait in terms of association with risk factors and adverse outcomes in population samples (Thapar & Cooper, 2016). Therefore, those with high ADHD trait levels in the general population are a relevant and important group to consider as well. Most studies investigating the associations between adult ADHD and neurocognitive impairments have been conducted in small clinical samples, comparing patients to healthy controls, with limited statistical power. Longitudinal research on small clinical samples has also only examined associations up to late adolescence (age 17) and has mostly focused on investigating neurocognitive test scores comparing those with persistent and remitted ADHD, indicating lower scores on neurocognitive tasks in both remitted and persistent ADHD groups compared to those without ADHD (Biederman et al., 2009; Cheung et al., 2016; Coghill, Hayward et al., 2014; McAuley et al., 2014; Riglin et al., 2022; van Lieshout et al., 2013). Therefore, it is not currently clear if the findings also extend to reported ADHD symptoms in the general population.

Even though neurocognitive deficits are not a defining criterion for ADHD, research has shown links between neurocognitive deficits, daily life functioning and quality of life (Sjöwall & Thorell, 2022). Those with ADHD and executive function deficits, on average, show greater impairment in occupational functioning and academic achievement compared to those with ADHD without EF deficits and to controls (without ADHD) (Barkley & Fischer, 2011; Barkley & Murphy, 2011; Halleland et al., 2019). Adults with ADHD from clinical samples who report more executive problems and impairment in daily living (self-report measures) were also found to be considerably more impaired for performance on the sustained attention and response inhibition tasks compared to controls (Grane et al., 2014; Salomone et al., 2020). However, again these associations have not been investigated in the general population.

Considering this lack of population based research into adult ADHD and neurocognitive profiles (Boonstra et al., 2005; Schoechlin & Engel, 2005; Willcutt et al., 2005), the aim of this study was to assess the pattern of associations between ADHD symptoms at age 25 and neurocognitive impairments assessed in childhood and adult life, in a UK population-based cohort. To meet this aim we examined whether:

(i) ADHD symptoms at age 25 were associated with ADHD cognitive task performance at age 25.
(ii) ADHD symptoms at age 25 were associated with ADHD cognitive task performance in childhood.

This was conducted both using total ADHD symptom scores and ADHD symptom domains of inattention and hyperactivity-impulsivity separately.

## Methods

### Sample

Data from the Avon Longitudinal Study of Parents and Children (ALSPAC) were used for this analysis. ALSPAC is a well-established longitudinal birth cohort study. Pregnant women resident in Avon, UK with expected dates of delivery between 1st April 1991 and 31st December 1992 were invited to take part in the study. The initial number of pregnancies enrolled was 14,541 (for these, at least one questionnaire has been returned or a “Children in Focus” clinic had been attended by 19/07/99). Of these initial pregnancies, “there was a total of 14,676 fetuses, resulting in 14,062 live births and 13,988 children who were alive at 1 year of age.” Part of this data was collected using REDCap (Harris et al., 2009, 2019). Please note that the study website contains details of all the data that is available through a fully searchable data dictionary and variable search tool: http://www.bristol.ac.uk/alspac/researchers/our-data/. Further details regarding establishment of the cohort and data collection can be seen in the Supplementary Materials and can be found elsewhere (Boyd et al., 2012; Fraser et al., 2013; Northstone et al., 2019).

Where individuals from the same family were included as participants, for example for multiple births, only data from the oldest sibling was included. Ethical approval for the study was obtained from the ALSPAC Ethics and Law Committee and the Local Research Ethics Committees. Informed consent for the use of data collected via questionnaires and clinics was obtained from participants following the recommendations of the ALSPAC Ethics and Law Committee at the time.

### Measures

#### ADHD Symptoms

ADHD symptoms were measured at age 25 years using the self-rated Barkley Adult ADHD Rating Scale (BAARS-IV) (Barkley, 2011). A parent-rated BAARS-IV was used for sensitivity analyses (Barkley, 2011). The BAARS-IV includes the 18 DSM-5 ADHD (American Psychiatric Association, 2013) items and assesses current ADHD symptoms using a four-point Likert scale. Clinically significant presence of each item was defined if it was endorsed as occurring “often” or “very often” in line with recommendations (Barkley, 2011). Total scores were derived for the two ADHD symptom domains of inattention and hyperactivity-impulsivity separately (ranges 0–9) and summed to generate a total symptom count (range 0–18).

ADHD symptoms in childhood were measured using the Development and Well-Being Assessment (DAWBA) (Goodman et al., 2000). The DAWBA is a structured diagnostic interview which was completed by parents as a questionnaire when their child was approximately 7 years old. The ADHD section of the DAWBA includes the 18 DSM-5 ADHD items (American Psychiatric Association, 2013) each rated on a four-point Likert scale. Each item rated as occurring “a lot more than others” was endorsed as being clinically significant, in line with previous research (Goodman et al., 2000). As in adulthood, total scores for the inattentive (range 0–9) and hyperactive-impulsive (range 0–9) domains were calculated, then summed to generate a total symptom count (range 0–18).

#### Cognitive Tasks

##### Measures of Attention

Attention was assessed at age 8 using the Sky Search task from the Test of Everyday Attention for Children (TEA-Ch) (Manly et al., 2001; Robertson et al., 1996). Participants were presented with an array of non-identical and identical spaceships and tasked with circling pairs of identical spaceships as quickly as possible. To control for the effect of motor speed, participants were asked to repeat the task without the non-identical pairs. Scores are derived by subtracting the mean time for the control condition (no non-identical pairs) from the experimental condition (includes non-identical pairs).

Attention in adulthood (age 25 years) was assessed online using the Sustained Attention to Response Task (SART) (Bellgrove et al., 2005). The SART is a measure of sustained attention that can predict everyday life attentional lapses and has previously been used to measure attention in ADHD clinical samples (Salomone et al., 2020; Shallice et al., 2002). The SART is a 5-min test where participants are presented with a sequence of 1 to 9 digits. Participants performed 225 trials, incorporating 25 No-Go trials. Participants were asked to respond to the presentation of certain digits (go-trials) whilst refraining from responding when others are presented (no-go trial), for example “Respond to the presentation of digits except when the digit ‘3.’ is presented.” Each correct response (regardless of whether it was a “Go” or “No-Go trial”) increased the total score by 1. The final total had 25 subtracted to prevent a total lack of response resulting in a score of 25 (i.e., each No-Go is correctly ignored).

Child and adult attention scores were transformed using log_10_, multiplied by minus one so that higher scores reflect better cognitive performance and subsequently standardized to mean = 0, Standard Deviation (*SD*) = 1 to aid interpretation.

##### Response Inhibition

Response inhibition in childhood (age 8) was assessed using the TEA-Ch Opposite Worlds task (Manly et al., 2001), which is a form of Stroop task (Stroop, 1935). Children were presented with a trail of digits “1” and “2” and were asked to read out the numbers in the “same world” (control) condition. In the “opposite world” condition children had to say the opposite number to the digit pointed to. After practice on each condition, four test conditions ran in the following order: a “same world” trial, two “opposite world” trials and a “same world” trial. The final score was derived by subtracting the mean time for the control condition (same worlds) from the experimental condition (opposite worlds), with lower scores reflecting better cognitive performance.

Response inhibition in adulthood (age 25) was assessed online using the Double Trouble task (also called Color—Word Remapping) (Hampshire et al., 2012; Metzler-Baddeley et al., 2016). This task is also a variation of the color-word Stroop Task (Stroop, 1935) whereby participants have to read the name of a color, printed in different colored ink. It differs through the inclusion of three words in every puzzle resulting in a more cognitively demanding task. Participants were presented with three words on a computer screen, one at the top and two at the bottom. Participants were required to click on the word at the bottom of the screen that correctly describes the color of the word at the top. To manipulate task difficulty, the congruency between font color and color meaning of the target and response words was varied. The main outcome measure was the total score which increased or decreased by 1 after each trial depending on whether the participant responded correctly. A score of 0 indicates an equal number of correct and incorrect responses, a score below 0 indicates more incorrect than correct whilst a score above 0 indicates more correct than incorrect responses produced. Higher scores (correct number of trials) on the response inhibition task are indicative of better cognitive performance.

Child response inhibition scores were multiplied by minus one so that both child and adult higher scores reflect better cognitive performance; all scores were standardized to mean = 0, *SD* = 1 to aid interpretation

Child cognitive measures were assessed at in-person clinic visits, whilst the adult assessments were conducted online. Data cleaning and removal of outliers for both attention (Sustained Attention to Response Task- SART) and response inhibition tasks (Double Trouble) was undertaken in accordance with guidance and instructions from Cambridge Brain Sciences (https://www.cambridgebrainsciences.com). Details are in Supplementary Material.

### Analyses

Primary analyses were conducted on those with cognitive data available for either task at age 25 (n=1543), with multiple imputation (MI) used to impute missing cognitive and ADHD symptom data. Full details of the multiple imputation strategy are given in the Supplementary Material.

#### Primary Analyses

Associations between ADHD symptoms and neurocognitive tasks across adulthood and childhood were examined using separate regressions with performance on each cognitive task as the outcome. Analysis was conducted first using total ADHD symptoms with subsequent analysis for the inattention and hyperactive/impulsive symptoms separately. At age 25, the device type used to complete the task (mobile phone, tablet, desktop computer as default) was included as a covariate. Sex was included as a covariate in all analyses.

#### Sensitivity Analyses Using Primary Sample

Sensitivity analyses were conducted by (i) using a categorical definition of ADHD in adulthood (symptoms above a cut-point of total score ≥5 at age 25) (American Psychiatric Association, 2013), (ii) using parent-rated adult ADHD total scores (measured using the Barkley Adult ADHD Rating Scale (Barkley, 2011)). These were conducted to examine if associations were similar using a binary definition of ADHD and for parent-rated adult ADHD. (iii) Lastly, to enable comparison with childhood ADHD symptoms in this cohort, we also tested associations between childhood ADHD and neurocognitive impairments.

#### Missing Data Approach

Analyses for addressing missing data were examined by (i) including anyone with available age 25 cognitive or (self-rated) ADHD data (*n* = 4,145), (ii) including anyone with available age 25 cognitive or (self-rated) ADHD data using multiple imputation combined with inverse probability weighting to the “full” ALSPAC sample (*n* = 14,692), and (iii) including only those with complete age 25 and childhood ADHD and cognitive task data (*n* = 792). Details of the multiple imputation strategy are given in the Supplementary Material.

## Results

### Descriptive

The primary sample with cognitive data at age 25 years (*n* = 1543) were 33.3% male. The mean self-rated adult ADHD symptom score using the Barkley scale was 1.42, (95% CI [1.28, 1.56]) and the mean childhood parent-rated ADHD symptom score using the DAWBA was 0.44, (95% CI [0.34, 0.55]) (Table 1). Within-domain longitudinal associations for ADHD symptom levels and cognitive domains are shown in the Supplementary Table 4. Associations between childhood ADHD and child cognitive impairments to enable comparison in the same sample were also tested and shown in Supplementary Table 5.

**Table 1.** Descriptive Statistics for Primary Variables (*n* = 1543).

	Mean/%	(95% CI)
ADHD symptoms:		
Adult total score ^a^	1.42	[1.28, 1.56]
Adult inattention ^a^	0.75	[0.67, 0.83]
Adult hyperactivity-impulsivity ^a^	0.70	[0.60, 0.74]
Child total score ^b^	0.44	[0.34, 0.55]
Child inattention ^b^	0.20	[0.15, 0.26]
Child hyperactivity-impulsivity ^b^	0.24	[0.18, 0.30]
Cognitive tasks:		
Adult attention^c^	−0.02	[−0.07, 0.03]
Adult response inhibition ^c^	−0.01	[−0.06, 0.04]
Child attention ^c^	−0.02	[−0.07, 0.04]
Child response inhibition ^c^	−0.02	[−0.08, 0.04]
Variables used in sensitivity analyses:		
Adult total score ≥5	10.34%	[8.82, 11.86]
Adult ADHD score: parent-rated	0.39	[0.28, 0.49]

a Barkley ADHD Scale self-rated at age 25 (range total score = 0–18, Inattention/hyperactivity- impulsivity = 0–9).

b DAWBA ADHD scale parent rated age 7 (range total score = 0–18, Inattention/hyperactivity- impulsivity = 0–9).

c Variables standardized prior to imputation.

### Associations Between Adult ADHD Symptoms and ADHD Cognitive Tasks in Adulthood

As shown in Table 2, higher ADHD total symptoms at age 25 were associated with lower scores on both ADHD cognitive tasks at age 25 years: a one SD unit increase in ADHD symptom score was linked to a mean 0.03 lower score for both cognitive tasks (attention *B* = −0.03, 95% CI [0.05, −0.01], *p* = .005; response inhibition *B* = −0.03, 95% CI [−0.05, −0.01], *p* = .002). When examining inattention and hyperactive-impulsivity scores separately, we observed similar patterns of associations.

**Table 2.** Associations Between ADHD Symptoms and ADHD Cognitive Tasks (*N* = 1,543).

	Total			Inattention			Hyperactivity-impulsivity		
	*B*	(95% CI)	*p*	*B*	(95% CI)	*p*	*B*	(95% CI)	*p*
Associations between ADHD symptoms and cognitive tasks at age 25^a^									
Attention	−0.03	[−0.05, −0.01]	.005	−0.04	[−0.08, −0.01]	.007	−0.05	[−0.09, −0.01]	.015
Response inhibition	−0.03	[−0.05, −0.01]	.002	−0.04	[−0.07, −0.01]	.008	−0.06	[−0.10, −0.02]	.003
Associations between ADHD symptoms at age 25 and cognitive tasks in childhood^b^									
Attention	−0.02	[−0.04, 0.00]	.125	−0.02	[−0.06, 0.01]	.246	−0.04	[−0.09, 0.01]	.081
Response inhibition	−0.02	[−0.04, 0.01]	.128	−0.03	[−0.06, 0.01]	.169	−0.04	[−0.08, 0.01]	.141

*Note*. Measures are standardized prior to imputation.

a Adjusted for sex and device type.

b Adjusted for sex.

### Associations Between Adult ADHD Symptoms and ADHD Cognitive Tasks in Childhood

Also shown in Table 2, the associations found between ADHD symptoms at age 25 with cognitive task performance in childhood were in the anticipated direction (lower performance in childhood cognitive task is associated with higher ADHD symptoms in adulthood) and of similar magnitudes as found for the cognitive tasks in adulthood (attention *B* = −0.02, 95% CI [0.04, 0.010] *p* = .125; response inhibition *B* = −0.02, 95% CI [−0.04, −0.01], *p* = .128).

Overall the results show that despite the differences in measurements and developmental period of childhood and adulthood, effect estimates were similar and small in magnitude.

### Sensitivity Analyses

Sensitivity analyses between dichotomized adult ADHD scores and cognitive tasks at age 25 showed a similar pattern of associations for the attention and response inhibition tasks but with wider confidence intervals (attention *B* = −0.05 (95% CI [−0.23, 0.13]), *p* = .592; response inhibition *B* = −0.17 (95% CI [−0.34, −0.00]), *p* = .049). We also observed this pattern when using parent-rated adult ADHD total scores, which showed moderate association with self-rated adult ADHD scores (*B* = 0.68, (95% CI [0.49, 0.88]), *p* = 2x10^-^10) (attention *B* = −0.03 (95% CI [−0.07, 0.02]), *p* = .270; response inhibition *B* = −0.07, 95% CI [−0.11, −0.02], *p* = .002). Each analysis using different approaches to missing data also found a similar pattern of results (Supplementary Table 7).

Sensitivity analyses between childhood ADHD and cognitive task performance at age 25 showed an association between higher ADHD scores in childhood and poorer performance on the response inhibition task at age 25, with weaker evidence for the attention task: a similar pattern as was observed for childhood cognitive task performance. Separate ADHD symptom domain analyses found associations of childhood inattention with both cognitive tasks at age 25, whereas for childhood hyperactivity-impulsivity there was only a weak association with attention at age 25 (Supplementary Table 5).

## Discussion

This study assessed associations between adult ADHD symptoms and childhood and adult cognitive performance, in a UK population-based sample. Higher self-rated ADHD symptoms at age 25 were associated with poorer performance on both cognitive tasks capturing ADHD-related functioning (attention and response inhibition) in adulthood. These associations were similar for both inattention and hyperactivity-impulsivity ADHD symptom domains.

Looking across time between ADHD symptoms at age 25 and cognitive tasks measured during childhood, the results showed weak evidence of association but a consistent pattern of elevated adult ADHD symptoms being associated with lower scores on the cognitive tasks across timepoints (childhood and age 25). Sensitivity analyses using an ADHD symptom cut-point and parent-rated adult ADHD symptoms also showed similar results which implied that neither the measurement scoring, nor rater, had an impact on the interpretation of our results.

Thus, our investigations indicate that associations between adult ADHD symptoms and neurocognitive impairments in a population sample are similar to those observed in studies of clinical samples. The estimates found overall were small in this population cohort. Childhood and adult clinical studies (Boonstra et al., 2005; Hervey et al., 2004; Mostert et al., 2015; Pievsky & McGrath, 2018; Schoechlin & Engel, 2005; Sonuga-Barke et al., 2010) have tended to report small to moderate effect sizes (Mostert et al., 2015). Despite the smaller estimates, the pattern of results were in the anticipated direction irrespective of the age, and cognitive task. Associations between ADHD symptoms and cognitive tasks were similar with each domain individually (i.e., the ADHD inattention domain with the attention cognitive task and the ADHD hyperactivity-impulsivity domain with the response inhibition task). This indicates that neurocognitive performance is likely similar across symptom subtypes, consistent with some previous clinical studies (Seidman, 2006; Tucha et al., 2009).

This study provides insight into associations between adult ADHD symptoms and neurocognitive impairment in a large population sample. Previous work has shown differences in adult ADHD symptom reporting, with adults in clinical studies tending to under-report their ADHD behaviors compared to other informants such as parents (Du Rietz et al., 2016; Mörstedt et al., 2015), whereas in population studies, some research suggest that individuals may report more symptoms compared to parent reports (Riglin et al., 2021). The findings of this study add to the clinical literature, suggesting that associations between adult ADHD symptoms (self-report) and neurocognitive impairments (in attention and response inhibition) are also found in a general population sample.

Findings should be considered in light of several limitations. Firstly, ALSPAC, like most longitudinal studies, suffers from non-random attrition, where individuals who are at higher risk of psychopathology are more likely to drop out (Martin et al., 2016; Taylor et al., 2018). There was considerable attrition from those initially recruited into the study to those included in our study, due to both lower questionnaire completion rates by age 25 and a sizable proportion of participants active at 25 not completing the cognitive task (n=1543 of *n* = 4145). Limiting the sample to those with just adult and cognitive task data likely resulted in this subsample not being representative of the wider population nor adequately capturing those at the extreme end of the ADHD symptom distribution—instead it is more akin to an opportunistic sample of adults at age 25, which may have resulted in less variance. Our findings were robust when using different approaches to missing data, including multiple imputation combined with IPW to the “full” ALSPAC sample, although these methods may also be subject to error and all assume data are missing at random (MAR). Secondly, the study focused on two cognitive measures associated with ADHD (attention and response inhibition). There are many different cognitive domains (e.g., vigilance and planning, reward dysregulation, processing speed) related to ADHD which have not yet been investigated in a population sample and future research could include these different neurocognitive domains. In addition, the cognitive tasks differed across time. This was necessary due to the developmental differences of the individuals when completing the measures, whilst the tasks were chosen to map onto the same domains. We also know that the presentation of ADHD differs over development. The magnitude of the associations across time and the direct comparison of within time associations should therefore be considered in light of these differences.

In summary, in a large, prospective longitudinal population sample, this study assessed the pattern of associations between ADHD symptoms at age 25 and neurocognitive impairments in both childhood and adulthood. Self-reported ADHD symptoms at age 25 were associated with neurocognitive impairments at a similar magnitude to ADHD symptoms reported in childhood. This indicates that, in the general population, ADHD symptoms reported at age 25 are reflected in cognitive impairments that are typical of ADHD. A similar pattern of association was also found for inattention and hyperactivity-impulsivity. The study suggests that neurocognitive impairments are similar for adult ADHD symptoms in the general population as those reported for studies in childhood, which is consistent with findings in adult ADHD clinical samples. Understanding more about neurocognitive impairment in adulthood can help shape the understanding of ADHD as a disorder across the lifespan. Further, identifying impairments in adults with ADHD through the assessment of neurocognitive deficits may also aid in the tailoring of suitable employment, training and personalized treatment options.

## Supplemental Material

sj-docx-1-jad-10.1177_10870547231201870 – Supplemental material for Young Adult ADHD Symptoms in the General Population and Neurocognitive Impairment Supplemental material, sj-docx-1-jad-10.1177_10870547231201870 for Young Adult ADHD Symptoms in the General Population and Neurocognitive Impairment by Sharifah Shameem Agha, Lucy Riglin, Rhian Carbury, Rachel Blakey, Amy Shakeshaft, Ajay K. Thapar, Kate Tilling, Stephan Collishaw, Evie Stergiakouli, Anita Thapar and Kate Langley in Journal of Attention Disorders
