# Supplementary material for: Young Adult ADHD Symptoms in the General Population and Neurocognitive Impairment
Source: J Atten Disord. 2023 Oct 21;28(1):89–98. doi: 10.1177/10870547231201870 (PMC10676027; doi:10.1177/10870547231201870)
Supplement: sj-docx-1-jad-10.1177_10870547231201870 – Supplemental material for Young Adult ADHD Symptoms in the General Population and Neurocognitive Impairment [file sj-docx-1-jad-10.1177_10870547231201870.docx]

**Version 2: 10 June 2025**

**Supplementary Material**

**The Avon Longitudinal Study of Parents and Children (ALSPAC)**

Pregnant women resident in Avon, UK with expected dates of delivery 1st April 1991 to 31st December 1992 were invited to take part in the study. The initial number of pregnancies enrolled is 14,541 (for these at least one questionnaire has been returned or a “Children in Focus” clinic had been attended by 19/07/99). Of these initial pregnancies, there was a total of 14,676 foetuses, resulting in 14,062 live births and 13,988 children who were alive at 1 year of age. When the oldest children were approximately 7 years of age, an attempt was made to bolster the initial sample with eligible cases who had failed to join the study originally. As a result, the total sample size for data collected after the age of seven is therefore 15,454 pregnancies, resulting in 15,589 foetuses. Of these 14,901 were alive at 1 year of age. Further details of the study, measures and sample can be found elsewhere (Boyd et al., 2013; Fraser et al., 2013; Northstone et al., 2019).

**Cognitive tasks**

In childhood, cognitive tasks were completed as part of a ‘Focus’ clinic at approximately age 8.5 years. In young-adulthood, participants who completed the ALSPAC questionnaire at age 25 years were invited to also complete cognitive tasks online.

Both the attention (Sustained Attention to Response Task- SART) and response inhibition tasks (Double Trouble) at age 25 were provided by Cambridge Brain Sciences ([https://www.cambridgebrainsciences.com](about:blank)) who adapted their existing tasks for online participation. Data cleaning and removal of outliers was undertaken in accordance with guidance and instructions from Cambridge Brain Sciences.

*Attention task (Sustained Attention to Response Task- SART):*

Scores of zero and below were considered outliers, representing participants who did not provide full effort or properly understand the task. These scores were removed for statistical analysis.

*Response inhibition task (Double Trouble):*

The following instructions are based on each criteria not being in the top or bottom 0.5% (i.e., 99% fall within these ranges) in a large sample:

- - Number of attempts is >0
  - Number of correct responses must be between >=11 and <=105
  - Number of errors must be between >=0 and <=37
  - Test duration (seconds) must be between >=89.5 and <=90.5
  - Final score must be between >= -5 and <=102

Additional measures of validity related to performance include problems such as 1) Congruent / Congruent (CC) problems, 2) Congruent / Incongruent (CI) problems, 3) Incongruent / Congruent problems (IC) and 4) Incongruent / Incongruent (II) problems.

Additional validity metrics for the Double Trouble Task include:

- % of CC problems answered correctly: >= 60% and <= 100%
- Average reaction time in seconds when attempting CC problems: >= .752 and <= 4.81
- Average reaction time in seconds when attempting CI problems: >= .778 and <= 6.15
- Average reaction time in seconds when attempting IC problems: >= .761 and <= 5.65
- Average reaction time in seconds when attempting II problems: >= .784 and <= 6.01

**Multiple imputation**

Individuals with cognitive data at age 25 years were included in our primary sample (N=1543). Both adult cognitive tasks were available for 86-94% of the sample, with self-report ADHD symptom counts available for 99%; childhood cognitive and ADHD symptom data were available for 73-79% of the sample (See Supplementary Table 1). There was a general trend for lower cognitive tasks scores and higher ADHD symptom counts to be associated with missing data in both adulthood and childhood (Supplementary Table 1).

Multiple imputation by chained equations (White et al., 2011) was used to impute missing cognitive task and ADHD symptom counts at each time-point whereby variables that were associated with missingness in these (Supplementary Table 2) and measures of these at different time-points (Supplementary Table 3) were included in an imputation model. The two ADHD symptoms domains inattention and hyperactivity-impulsivity were imputed separately as ordinal variables and then summed post-imputation to generate a total symptom count. We generated 250 imputed datasets, which was estimated to be a sufficient number of imputations to ensure that standard errors would not change considerably if the data were imputed again (the recommended 2-stage quadratic rule based on the initial imputation of 250 datasets suggested 3-32 imputations were needed for the primary analyses)(von Hippel, 2018). Estimates were combined across imputed datasets using Rubin’s rules (White et al., 2011).

**Missing data sensitivity analyses**

*Including anyone with available cognitive data and self-rated ADHD data*

Individuals with cognitive data or ADHD data (self-report) at age 25 years were included in our sensitivity sample (N=4145). Associations with missingness within the sample are shown in Supplementary Table 6. Multiple imputation by chained equations was used to generate 250 imputed datasets using the same procedure and variables included in the primary imputation (the 2-stage quadratic rule suggested 30-141 imputations were needed for the primary analyses) (von Hippel, 2018).

Results are shown in Supplementary Table 7 and are very similar to those observed for the primary sample.

*Including anyone with available cognitive data self-rated ADHD data: with inverse probability weighting to the “full” ALSPAC sample*

Multiple imputation for individuals with cognitive data or ADHD data (self-report) at age 25 years (N=4145) was repeated including inverse probability weights (Seaman et al., 2012) to the “full” ALSPAC sample (i.e. including those without cognitive or ADHD data at age 25 years, N=14692). Inverse probability weighting (IPW) was used to generate weights derived from a logistic regression analysis of missing age 25 data for the measures assessed in or soon after pregnancy with minimal missingness that were that showed independent association with missing data (see Supplementary Table 8). Missing data on indicators used to derive weights were singly imputed as the modal or mean value (all indicators had <20% of values missing). The Hosmer-Lemeshow test was used to assess the fit of the missingness model and did not indicate poor fit (Hosmer-Lemeshow χ2(8)=11.94, p=0.15). Weights were stabilized whereby the numerator was the probability of inclusions in the sample (i.e. 4145/14692) (Sayon-Orea et al., 2020). For the included sample (N=4145) weights ranged from 0.43 to 7.76. Again, multiple imputation by chained equations was used to generate 250 imputed datasets using the same procedure and variables (plus the stabilized wight) included in the primary imputation (the 2-stage quadratic rule suggested 47-118 imputations were needed for the primary analyses) (von Hippel, 2018). A robust estimator was used due to uncertainty in weights (Seaman et al., 2012).

Results are shown in Supplementary Table 7 and are similar to those observed for the primary sample.

*Complete cases*

Analyses using complete cases included individuals with complete cognitive and ADHD symptom data at ages 7 and 25 years (N=792). Results are shown in Supplementary Table 7 and are similar to those observed for the primary sample.

**References**

Boyd, A., Golding, J., Macleod, J., Lawlor, D. A., Fraser, A., Henderson, J., Molloy, L., Ness, A., Ring, S., & Smith, G. D. (2013). Cohort Profile: the ’children of the 90s’--the index offspring of the Avon Longitudinal Study of Parents and Children. *International Journal of Epidemiology*, *42*(1), 111–127. https://doi.org/10.1093/IJE/DYS064

Fraser, A., Macdonald-wallis, C., Tilling, K., Boyd, A., Golding, J., Davey smith, G., Henderson, J., Macleod, J., Molloy, L., Ness, A., Ring, S., Nelson, S. M., & Lawlor, D. A. (2013). Cohort Profile: the Avon Longitudinal Study of Parents and Children: ALSPAC mothers cohort. *International Journal of Epidemiology*, *42*(1), 97–110. https://doi.org/10.1093/IJE/DYS066

Northstone, K., Lewcock, M., Groom, A., Boyd, A., Macleod, J., Timpson, N., & Wells, N. (2019). The Avon Longitudinal Study of Parents and Children (ALSPAC): an update on the enrolled sample of index children in 2019. *Wellcome Open Research*, *4*. https://doi.org/10.12688/WELLCOMEOPENRES.15132.1

Sayon-Orea, C., Moreno-Iribas, C., Delfrade, J., Sanchez-Echenique, M., Amiano, P., Ardanaz, E., Gorricho, J., Basterra, G., Nuin, M., & Guevara, M. (2020). Inverse-probability weighting and multiple imputation for evaluating selection bias in the estimation of childhood obesity prevalence using data from electronic health records. *BMC Medical Informatics and Decision Making*, *20*(1). https://doi.org/10.1186/S12911-020-1020-8

Seaman, S. R., White, I. R., Copas, A. J., & Li, L. (2012). Combining multiple imputation and inverse-probability weighting. *Biometrics*, *68*(1), 129–137. https://doi.org/10.1111/J.1541-0420.2011.01666.X

von Hippel, P. T. (2018). How Many Imputations Do You Need? A Two-stage Calculation Using a Quadratic Rule. *Https://Doi.Org/10.1177/0049124117747303*, *49*(3), 699–718. https://doi.org/10.1177/0049124117747303

White, I. R., Royston, P., & Wood, A. M. (2011). Multiple imputation using chained equations: Issues and guidance for practice. *Statistics in Medicine*, *30*(4), 377–399. https://doi.org/10.1002/SIM.4067

| Supplementary **Table 1.** Associations between ADHD cognitive tasks and symptom counts and missing ADHD cognitive task and symptom count data | | | | |
| --- | --- | --- | --- | --- |
|  | Available data | | Association with available cognitive/ADHD data in primary sample^*^ | |
|  | N | % | Adult | Child |
| Cognitive tasks |  |  |  |  |
| Adult attention | 1328 | 86% | OR=0.70 (95% CI=0.57-0.87) | OR=0.91 (95% CI=0.81-1.02) |
| Adult response inhibition | 1454 | 94% | OR=0.64 (95% CI=0.56-0.73) | OR=0.89 (95% CI=0.80-0.98) |
| Child attention | 1124 | 73% | OR=0.96 (95% CI=0.82-1.11) | OR=1.15 (95% CI=0.97-1.37) |
| Child response inhibition | 1137 | 74% | OR=0.94 (95% CI=0.81-1.09) | OR=1.21 (95% CI=1.03-1.43) |
| ADHD symptom counts |  |  |  |  |
| Adult inattention | 1535 | 99% | OR=1.00 (95% CI=0.93-1.08) | OR=1.03 (95% CI= 0.97-1.10) |
| Adult hyperactivity-impulsivity | 1535 | 99% | OR=1.05 (95% CI=0.96-1.14) | OR=1.08 (95% CI= 1.00-1.16) |
| Child inattention | 1213 | 79% | OR=1.03 (95% CI= 0.90-1.19) | OR=1.07 (95% CI= 0.94-1.22) |
| Child hyperactivity-impulsivity | 1207 | 78% | OR=1.01 (95% CI= 0.89-1.16) | OR=1.08 (95% CI= 0.96-1.22) |
| Total possible N=1543. ^*^Missing on either subscale/domain. Adult self-report ADHD data. Cognitive test standardised to mean = 0 SD = 1. | | | | |

| Supplementary **Table 2.** Additional measures included in the imputation model that were associated with missing ADHD cognitive task and symptom count data | | | | |
| --- | --- | --- | --- | --- |
|  | Available data | | Association with available cognitive/ADHD data in primary sample^*^ | |
|  | N | % | Adult | Child |
| Male sex | 1543 | 100% | OR=0.51  (95% CI=0.38-0.68) | OR=0.66 (95% CI=0.52-0.82) |
| Home ownership | 1396 | 90% | OR=0.59  (95% CI=0.42-0.83) | OR=0.44 (95% CI=0.33-0.60) |
| Smoked during pregnancy^**^ | 1363 | 88% | OR=1.69  (95% CI=1.15-2.49) | OR=1.86 (95% CI=1.32-2.63) |
| Maternal history of depression | 1396 | 90% | OR=1.03  (95% CI=0.61-1.74) | OR=1.66 (95% CI=1.09-2.54) |
| Maternal age at birth | 1436 | 93% | OR=0.95  (95% CI=0.92-0.98) | OR=0.93 (95% CI=0.90-0.95) |
| Maternal highest education | 1394 | 90% | OR=0.77  (95% CI=0.69-0.86) | OR=0.74 (95% CI=0.67-0.82) |
| Total possible N=1543. ^*^Missing on either subscale/domain. ^**^In last 2 months of pregnancy. Adult self-report ADHD data. | | | | |

| Supplementary **Table 3.** Additional measures of ADHD cognitive tasks / symptom counts included in the imputation model | | | | | | | | | | |
| --- | --- | --- | --- | --- | --- | --- | --- | --- | --- | --- |
|  | Available data | | Correlation with cognitive/ADHD data in primary sample | | | | | | | |
|  | N | % | Adult A | Adult RI | Child A | Child RI | Adult In | Adult HI | Child In | Child HI |
| Cognitive tasks |  |  |  |  |  |  |  |  |  |  |
| A: age 11 | 1186 | 77% | 0.10 | 0.11 | 0.38 | 0.13 | -0.00 | -0.00 | -0.07 | 0.00 |
| RI: age 11 | 1143 | 74% | 0.14 | 0.25 | 0.20 | 0.30 | -0.04 | -0.06 | -0.06 | 0.00 |
| ADHD symptom counts |  |  |  |  |  |  |  |  |  |  |
| In: age 10 | 1254 | 81% | -0.02 | -0.05 | -0.13 | -0.05 | 0.08 | 0.09 | 0.45 | 0.45 |
| HI: age 10 | 1254 | 81% | -0.00 | -0.05 | -0.05 | -0.03 | 0.08 | 0.10 | 0.46 | 0.70 |
| In: age 13 | 1210 | 78% | -0.04 | -0.03 | -0.07 | -0.06 | 0.13 | 0.14 | 0.29 | 0.25 |
| HI: age 13 | 1210 | 78% | -0.03 | -0.05 | 0.01 | -0.03 | 0.15 | 0.19 | 0.23 | 0.44 |
| In: age 15 | 990 | 64% | -0.06 | -0.07 | -0.04 | -0.07 | 0.13 | 0.12 | 0.35 | 0.35 |
| HI: age 15 | 994 | 64% | 0.01 | -0.02 | -0.08 | -0.04 | 0.08 | 0.10 | 0.18 | 0.34 |
| In: parent-report age 25 | 1044 | 68% | -0.05 | -0.06 | 0.01 | -0.05 | 0.37 | 0.34 | 0.23 | 0.30 |
| HI: parent-report age 25 | 1043 | 68% | -0.02 | -0.12 | -0.01 | -0.10 | 0.26 | 0.29 | 0.14 | 0.28 |
| Total possible N=1543. SA = sustained attention, RI = response inhibition, In = inattention, HI = Hyperactivity-impulsivity. Adult self-report ADHD data. Cognitive test standardised to mean = 0 SD = 1. Age in years. | | | | | | | | | | |

| Supplementary **Table 4:** Within-domain associations between childhood and adult ADHD symptoms and cognitive domains (primary sample) N=1543 | | | |
| --- | --- | --- | --- |
| ADHD symptom score | B | (95% CI) | p |
| Total | 0.15 | (0.05, 0.24) | 0.003 |
| Inattention | 0.10 | (-0.01, 0.21) | 0.070 |
| Hyperactivity-impulsivity | 0.14 | (0.06, 0.22) | 5x10^-05^ |
| Cognitive Tasks |  |  |  |
| Attention | 0.08 | (0.02, 0.15) | 0.011 |
| Response Inhibition | 0.22 | (0.16, 0.27) | 5x10^-13^ |
| *adjusted for sex and device type, **adjusted for sex | | | |

| Supplementary **Table 5:** Associations between ADHD symptoms in childhood and ADHD cognitive tasks at age 8 and 25 years* | | | | | | | | | | | | | |
| --- | --- | --- | --- | --- | --- | --- | --- | --- | --- | --- | --- | --- | --- |
|  | Primary sample | | | Missing data sensitivity analyses: | | | | | | | | | |
|  | Using MI to impute missing data for those with cognitive data available for either task at age 25 (N=1543) | | | Using MI for those with cognitive/ADHD symptom data at age 25 (N=4145) | | | Using MI for those with cognitive/ADHD symptom data at age 25 combined with IPW to “full” ALSPAC sample (N=14692) | | | Using complete cases of those with cognitive and ADHD symptom data at age 7 and 25 (N=792) | | | |
| ***Associations between ADHD symptoms and cognitive tasks in childhood**** | | | | | | | | | | | | | |
|  | Total | Inattention | Hyperactivity-impulsivity | Total | Inattention | Hyperactivity-impulsivity | Total | Inattention | Hyperactivity-impulsivity | Total | Inattention | Hyperactivity-impulsivity |  |
| Attention | B=-0.01  (-0.04, 0.03)  p=0.679 | B=-0.05  (-0.11, 0.02)  p=0.165 | B=0.02  (-0.04, 0.08)  p=0.569 | B=-0.02  (-0.04, 0.00)  p=0.096 | B=-0.06  (-0.10, 0.02)  p=0.03 | B=-0.00  (-0.04, 0.04)  p=0.934 | B=-0.02  (-0.04, 0.01)  p=0.255 | B=-0.05  (-0.11, 0.0)  p=0.034 | B=-0.00  (-0.04, 0.05)  p=0.956 | B=0.03  (-0.01, 0.07)  p=0.190 | B=0.02  (-0.05, 0.10)  p=0.570 | B=0.06  (-0.01, 0.013)  p=0.081 |  |
| Response inhibition | B=-0.05  (-0.08, -0.01)  p=0.009 | B=-0.11  (-0.17, -0.04)  p=0.002 | B=-0.06  (-0.12, 0.00)  p=0.064 | B=-0.04  (-0.06, -0.02)  p=3x10^-04^ | B=-0.09  (-0.13, -0.05)  p=8x10^-06^ | B=-0.04  (-0.07, 0.00)  p=0.047 | B=-0.45  (-0.07, -0.01)  p=0.007 | B=-0.10  (-0.15, -0.04)  p=3x10^-04^ | B=-0.03  (-0.08, 0.01)  p=0.149 | B=-0.04  (-0.08, -0.01)  p=0.093 | B=-0.09  (-0.17, -0.01)  p=0.027 | B=-0.03  (-0.11, 0.04)  p=0.396 |  |
| ***Associations between ADHD symptoms in childhood cognitive tasks at age 25 years***** | | | | | | | | | | | | | |
|  | Total | Inattention | Hyperactivity-impulsivity | Total | Inattention | Hyperactivity-impulsivity | Total | Inattention | Hyperactivity-impulsivity | Total | Inattention | Hyperactivity-impulsivity |  |
| Attention | B=-0.03  (-0.06, 0.01) p=0.112 | B=-0.06  (-0.13, 0.00) p=0.042 | B= -0.03  (-0.08, 0.03) p=0.361 | B=-0.03  (-0.06, 0.01) p=0.130 | B=-0.06  (-0.13, 0.00) p=0.062 | B=-0.03  (-0.09, 0.03) p=0.367 | B=-0.03  (-0.06, 0.01) p=0.198 | B=-0.07  (-0.14, 0.01) p=0.080 | B=-0.02  (-0.09, 0.04) p=0.514 | B=-0.04  (-0.11, 0.03) p=0.225 | B=-0.07  (-0.15, 0.003) p=0.060 | B=-0.01  (-0.08, 0.06) p=0.727 |  |
| Response inhibition | B=-0.04  (-0.07, -0.01) p=0.007 | B=-0.09  (-0.15, -0.03) p=0.003 | B=-0.06  (-0.11, -0.01) p=0.020 | B=-0.05  (-0.08, -0.01) p=0.005 | B=-0.09  (-0.15, -0.03) p=0.003 | B=-0.07  (-0.13, -0.01) p=0.018 | B=-0.05  (-0.09, -0.01)  p=0.010 | B=-0.10  (-0.17, -0.02) p=0.010 | B=-0.08  (-0.15, -0.01)  p=0.021 | B=-0.04  (-0.11, -0.03) p=0.299 | B=-0.05  (-0.12, 0.02) p=0.181 | B=-0.02  (-0.09, 0.05) p=0.581 |  |
| *adjusted for sex, **adjusted for sex and device type  MI = Multiple imputation, IPW = Inverse probability weighting, B= beta, 95% confidence interval in parentheses | | | | | | | | | | | | |  |

| Supplementary **Table 6.** Associations with missing ADHD cognitive task and symptom count data in sensitivity sample | | | | |
| --- | --- | --- | --- | --- |
|  | Available data | | Association with available cognitive/ADHD data in sensitivity sample^*^ | |
|  | N | % | Adult | Child |
| Cognitive tasks |  |  |  |  |
| Adult attention | 1328 | 32% | OR=0.70 (95% CI=0.57-0.87) | OR=0.91 (95% CI=0.81-1.02) |
| Adult response inhibition | 1454 | 35% | OR=0.64 (95% CI=0.56-0.73) | OR=0.89 (95% CI=0.80-0.98) |
| Child attention | 2977 | 72% | OR=0.96 (95% CI=0.89-1.03) | OR=1.01 (95% CI=0.91-1.13) |
| Child response inhibition | 3001 | 72% | OR=0.91 (95% CI=0.84-0.99) | OR=1.06 (95% CI=0.96-1.17) |
| ADHD symptom counts |  |  |  |  |
| Adult inattention | 4137 | >99% | OR=1.00 (95% CI=0.96-1.04) | OR=1.06 (95% CI= 1.02-1.09) |
| Adult hyperactivity-impulsivity | 4137 | >99% | OR=1.06 (95% CI=1.01-1.11) | OR=1.09 (95% CI= 1.05-1.14) |
| Child inattention | 3195 | 77% | OR=1.07 (95% CI= 0.99-1.15) | OR=1.16 (95% CI= 1.04-1.19) |
| Child hyperactivity-impulsivity | 3192 | 77% | OR=1.05 (95% CI= 0.98-1.13) | OR=1.11 (95% CI= 1.04-1.19) |
| Additional variables |  |  |  |  |
| Male sex | 4145 | 100% | OR=0.86  (95% CI=0.75-0.99) | OR=0.82 (95% CI=0.71-0.93) |
| Home ownership | 3737 | 90% | OR=0.77  (95% CI=0.63-0.94) | OR=0.45 (95% CI=0.38-0.54) |
| Smoked during pregnancy^**^ | 3625 | 87% | OR=1.43  (95% CI=1.14-1.79) | OR=1.90 (95% CI=1.45-2.21) |
| Maternal history of depression | 3715 | 90% | OR=0.89  (95% CI=0.67-1.18) | OR=1.57 (95% CI=1.20-2.05) |
| Maternal age at birth | 3849 | 93% | OR=0.97  (95% CI=0.95-0.98) | OR=0.93 (95% CI=0.92-0.94) |
| Maternal highest education | 3714 | 90% | OR=0.79  (95% CI=0.75-0.84) | OR=0.73 (95% CI=0.69-0.77) |
| Total possible N=4145. ^*^Missing on either subscale/domain. ^**^In last 2 months of pregnancy. Adult self-report ADHD data. Cognitive test standardised to mean = 0 SD = 1. | | | | |

| Supplementary **Table 7: Results of primary analyses and missing data sensitivity analyses** | | | | | | | | | | | | | |
| --- | --- | --- | --- | --- | --- | --- | --- | --- | --- | --- | --- | --- | --- |
|  | Primary sample | | | Missing data sensitivity analyses: | | | | | | | | | |
|  | Using MI to impute missing data for those with cognitive data available for either task at age 25 (N=1543) | | | Using MI for those with cognitive/ADHD symptom data at age 25 (N=4145) | | | Using MI for those with cognitive/ADHD symptom data at age 25 combined with IPW to “full” ALSPAC sample (N=14692) | | | Using complete cases of those with cognitive and ADHD symptom data at age 7 and 25 (N=792) | | | |
| ***Associations between ADHD symptoms and cognitive tasks*** | | | | | | | | | | | | | |
|  | Total | Inattention | Hyperactivity-impulsivity | Total | Inattention | Hyperactivity-impulsivity | Total | Inattention | Hyperactivity-impulsivity | Total | Inattention | Hyperactivity-impulsivity |  |
| Attention | B=-0.03  (-0.05, -0.01) p=0.005 | B=-0.04  (-0.08, -0.01) p=0.007 | B=-0.05  (-0.09, -0.01) p=0.015 | B=-0.03  (-0.05, -0.01)  p=0.017 | B=-0.04  (-0.08, -0.01) p=0.010 | B=-0.04  (-0.09, 0.00) p=0.051 | B=-0.03  (-0.05, -0.00)  p=0.028 | B=-0.05  (-0.09, -0.01)  p=0.019 | B=-0.05  (-0.10, 0.00)  p=0.069 | B=-0.07 (-0.14, -0.001) p=0.048 | B=-0.06 (-0.13, 0.01) p=0.085 | B=-0.07  (-0.14, -0.003) p=0.061 |  |
| Response inhibition | B=-0.03 (-0.05, -0.01), p= 0.002 | B=-0.04 (-0.07, -0.01), p=0.008 | B=-0.06 (-0.10, -0.02), p=0.003 | B=-0.03  (-0.05, -0.01) p=0.003 | B=-0.05  (-0.08, -0.01) p=0.006 | B=-0.06  (-0.10, -0.02) p=0.005 | B=-0.03  (-0.06, -0.01)  p=0.005 | B=-0.05  (-0.09, -0.01)  p=0.011 | B=-0.07  (-0.12, -0.02)  p=0.006 | B=-0.06  (-0.13, 0.01) p=0.074 | B=-0.05  (-0.11, 0.02) p=0.200 | B=-0.07  (-0.14, -0.003) p=0.040 |  |
| ***Associations between ADHD symptoms at age 25 and cognitive tasks in childhood*** | | | | | | | | | | | | | |
|  | Total | Inattention | Hyperactivity-impulsivity | Total | Inattention | Hyperactivity-impulsivity | Total | Inattention | Hyperactivity-impulsivity | Total | Inattention | Hyperactivity-impulsivity |  |
| Attention | B=-0.02  (-0.04, 0.00) p=0.125 | B=-0.02  (-0.06, 0.01) p=0.246 | B=-0.04 (-0.09, 0.01) p=0.081 | B=-0.00  (-0.02, 0.01) p=0.496 | B=-0.01  (-0.03, 0.02) p=0.649 | B=-0.01  (-0.04, 0.01) p=0.397 | B=-0.001  (-0.02, 0.01)  p=0.480 | B=-0.01  (-0.04, 0.02) p=0.508 | B=-0.01  (-0.04, 0.02) p=0.508 | B=-0.06  (-0.12, 0.01) p=0.113 | B=-0.03  (-0.10, 0.04) p=0.354 | B=-0.07  (-0.14, -0.004) p=0.037 |  |
| Response inhibition | B=-0.02  (-0.04, 0.01) p=0.128 | B=-0.03  (-0.06, 0.01) p=0.169 | B=-0.04 (-0.08, -0.01) p=0.141 | B=-0.01  (-0.03, 0.00) p=0.087 | B=-0.01  (-0.04, 0.01) p=0.221 | B=-0.03  (-0.05, -0.00) p=0.044 | B=-0.02  (-0.03, -0.00) p=0.087 | B=-0.02  (-0.05, 0.01) p=0.174 | B=-0.03  (-0.07, 0.00) p=0.056 | B=-0.02  (-0.09, 0.05) p=0.534 | B=-0.01  (-0.08, 0.06) p=0.882 | B=-0.04  (-0.11, 0.03) p=0.275 |  |
|  | | | | | | | | | | | | | |
| MI = Multiple imputation, IPW = Inverse probability weighting, B= beta, 95% confidence interval in parentheses | | | | | | | | | | | | |  |

| Supplementary **Table 8.** Associations with missing ADHD cognitive task and symptom count data in the “full” ALSPAC sample | | | | |
| --- | --- | --- | --- | --- |
|  | Available data | | Association with exclusion from sensitivity sample^*^ | |
|  | N | % | Univariable analyses | Final multivariable analysis for IPW |
| Male sex | 14692 | 100% | OR=2.71  (95% CI=2.51-2.92) | OR=2.87 (95% CI=2.66-3.11) |
| Home ownership | 12858 | 88% | OR=0.42 (95% CI=0.38-0.47) | OR=0.68 (95% CI=0.61-0.75) |
| Smoked during pregnancy^**^ | 11689 | 80% | OR=2.03 (95% CI=1.82-2.27) | - |
| Maternal history of depression | 12294 | 84% | OR=1.69 (95% CI=1.46-1.96) | OR=1.32 (95% CI=1.13-1.55) |
| Maternal age at birth | 13788 | 94% | OR=0.93 (95% CI=0.92-0.94) | OR=0.94 (95% CI=0.93-0.95) |
| Maternal highest education | 12252 | 83% | OR=0.69 (95% CI=0.67-0.72) | OR=0.75 (95% CI=0.73-0.78) |
| Gestation | 13788 | 94% | OR=0.96 (95% CI=0.94-0.98) | OR=0.98 (95% CI=0.96-1.00) |
| Birth weight | 13615 | 93% | OR=1.00 (95% CI=1.00-1.00) | - |
| Parity | 12757 | 87% | OR=1.17 (95% CI=1.12-1.22) | OR=1.15 (95% CI=1.10-1.21) |
| Total possible N=14692. ^*^Missing on either subscale/domain. ^**^In last 2 months of pregnancy. | | | | |
